# The Activation of Endothelial Cells Relies on a Ferroptosis-Like Mechanism: Novel Perspectives in Management of Angiogenesis and Cancer Therapy

**DOI:** 10.3389/fonc.2021.656229

**Published:** 2021-05-10

**Authors:** Filipa Lopes-Coelho, Filipa Martins, Ana Hipólito, Cindy Mendes, Catarina O. Sequeira, Rita F. Pires, António M. Almeida, Vasco D. B. Bonifácio, Sofia A. Pereira, Jacinta Serpa

**Affiliations:** ^1^ Unidade de Investigação em Patobiologia Molecular (UIPM), Instituto Português de Oncologia de Lisboa Francisco Gentil (IPOLFG), Lisboa, Portugal; ^2^ CEDOC, Chronic Diseases Research Centre, NOVA Medical School, Faculdade de Ciências Médicas, Universidade NOVA de Lisboa, Lisboa, Portugal; ^3^ iBB-Institute for Bioengineering and Biosciences, Instituto Superior Técnico, Universidade de Lisboa, Lisboa, Portugal; ^4^ Hematology, Hospital da Luz, Lisboa, Portugal

**Keywords:** angiogenesis, ferroptosis, oxidative stress, lipid peroxidation, endothelial cell hyperactivation, propranolol, polyurea dendrimers, tumor vasculature stabilizers

## Abstract

The activation of endothelial cells (ECs) is a crucial step on the road map of tumor angiogenesis and expanding evidence indicates that a pro-oxidant tumor microenvironment, conditioned by cancer metabolic rewiring, is a relevant controller of this process. Herein, we investigated the contribution of oxidative stress-induced ferroptosis to ECs activation. Moreover, we also addressed the anti-angiogenic effect of Propranolol. We observed that a ferroptosis-like mechanism, induced by xCT inhibition with Erastin, at a non-lethal level, promoted features of ECs activation, such as proliferation, migration and vessel-like structures formation, concomitantly with the depletion of reduced glutathione (GSH) and increased levels of oxidative stress and lipid peroxides. Additionally, this ferroptosis-like mechanism promoted vascular endothelial cadherin (VE-cadherin) junctional gaps and potentiated cancer cell adhesion to ECs and transendothelial migration. Propranolol was able to revert Erastin-dependent activation of ECs and increased levels of hydrogen sulfide (H_2_S) underlie the mechanism of action of Propranolol. Furthermore, we tested a dual-effect therapy by promoting ECs stability with Propranolol and boosting oxidative stress to induce cancer cell death with a nanoformulation comprising selenium-containing chrysin (SeChry) encapsulated in a fourth generation polyurea dendrimer (SeChry@PURE_G4_). Our data showed that novel developments in cancer treatment may rely on multi-targeting strategies focusing on nanoformulations for a safer induction of cancer cell death, taking advantage of tumor vasculature stabilization.

## Introduction

Tumor blood vessels are essential to provide nutrients and oxygen to cancer cells and for the elimination of waste products. Besides the promotion of tumor growth, the neovasculature acts as a gatekeeper for tumor cell invasion and metastasis (1). In an ideal scenario, the tackling of tumor angiogenesis would be an efficient anti-cancer approach, yet, so far, the anti-angiogenic therapies have shown a lack of efficacy and drug resistance (2, 3).

During angiogenesis, the balance between pro-angiogenic factors (e.g., vascular endothelial growth factor - VEGF, fibroblast growth factors - FGFs, and angiopoietins - ANGPTs) and anti-angiogenic factors (e.g., endostatin, thrombospondin, and angiostatin) plays a vital role in the regulation of the angiogenic switch, a process characterized by the activation of the quiescent endothelial cells (ECs) to form new blood vessels (4). However, contrarily to physiological angiogenesis, cancer neovessels are unorganized and leakier, suggesting that an imbalance in pro- and anti-angiogenic factors or the activation of unknown signaling pathways triggers a hyperactivation of the angiogenic switch and further unstable cancer neovessel formation (5).

Still, for both physiological and pathological angiogenesis the oxidative stress represents a pro-angiogenic stimulus (6, 7). Cancer neoangiogenesis seems to be more responsive to oxidative stress than physiological angiogenesis because the metabolic remodeling of malignant cells and tumor-associated stromal cells contributes to the generation of a pro-oxidative tumor microenvironment (8). At a molecular level, reactive oxygen species (ROS) inhibit PHDs (prolyl hydroxylases) leading to hypoxia-inducible factor 1α (HIF1α) stabilization, and consequently to the transcription of VEGF and other pro-angiogenic factors (6, 7).

The oxidative stress-dependent generation of lipid peroxides underlies ferroptosis, a recently discovered process of programmed cell death. In this process, iron ions (Fe^2+^) promote lipid oxidation in a Fenton-like reaction, increasing ROS levels alongside with intracellular glutathione (GSH) depletion, leading to the impairment of the activity of glutathione peroxidase 4 (GPX4), a GSH-dependent hydroperoxidase responsible for the scavenging of lipid peroxides (9, 10). The ROS-induced lipid peroxidation damages membranar phospholipids directly and can also act as cell death signal. Recent observations have shown that ferroptosis is not strictly a cell death type; it can also be associated with the regulation of biological and pathophysiological processes, including carcinogenesis (9, 11). Ferroptosis can be induced by angiopoietin-like 4 (ANGPTL4), a potent angiogenic mediator that activates the TAZ-ANGPTL4-NOX2 (transcriptional coactivator with PDZ-binding motif-ANGPTL4-NADPH oxidase 2) axis. This axis is responsible for the activation of NADPH oxidase 2 (NOX2), that induces the superoxide radical generation, which in turn acts as an activator of ferroptosis (12), suggesting a correlation between ferroptosis and angiogenesis induction.

In the last years, Propranolol, a liposoluble non-selective β-blocker, without an intrinsic activity (13) and with a well-known membrane stabilizing effect (14), firstly indicated as an anti-hypertensive drug was repurposed as a first-line therapy for vascular tumors, such as infantile hemangiomas and cavernomas (15, 16). Furthermore, breast cancer patients exposed to β-blockers prior or after diagnosis had a better disease prognosis and less metastases (17–19). So far, it is recognized that the impairment of angiogenesis by Propranolol involves the downregulation of VEGF and FGF expression, and consequently the inhibition of mitogen-activated protein kinase (MAPK) signaling pathway (20, 21). Although it seems to be independent of its β-blocker action (22), the specific mechanism(s) of action by which Propranolol affects ECs and angiogenesis remains to be clarified. A paper from Sasaki’s team demonstrated that Propranolol downregulates the expression of ANGPTL4 in hemangioma cells (22). ANGPTL4 regulates angiogenesis in a context-dependent manner, acting as a pro- or as an anti-angiogenic factor (23, 24).

Apart from the inhibition of ANGPTL4 expression and consequent abrogation of NOX2 activation, Propranolol could also interfere with ferroptosis through the inhibition of cytochrome P450 (CYP450) enzymes, whose activity promotes ROS and lipid peroxidation (25, 26). Propranolol is a substrate of CYP450s (27–29), acting as an inhibitor of CYP2D6 (30), CYP2C19 (31) and CYP1A2 (27, 32) isoenzymes. Recently, Mishima et al. identified the anti-ferroptotic properties of Propranolol, which promoted lipid peroxyl radicals scavenging in a β1 activity-independent manner (29). Although the mechanism underlying the anti-angiogenic effect of Propranolol is not clear, it was shown that it does not involve apoptosis (33).

Here, we investigated if ECs activation needed for angiogenesis might be triggered by a ferroptosis-like mechanism. In addition, we disclosed if this mechanism contributes to the anti-angiogenic effect of Propranolol. Finally, we made a pilot proof of concept approach to address the hypothesis that a promising chemotherapeutic strategy could combine inducers of cell death and ECs stabilizers.

## Material and Methods

### Cell Culture

Three different batches corresponding to three different-original donors of Human umbilical vein endothelial cells (HUVECs: CRL-1730, ATCC) were used. HUVECs were cultured in Endothelial Cell Growth Basal Medium-2 (EBM-2: CC-3156, Lonza, Bioscience) supplemented with EGM-2 SingleQuots Supplements (CC-4176, Lonza, Bioscience). All experiments were performed until the passage 10. Triple-negative breast cancer (MDA-MB-231: HTB-26™, ATCC) were used as tumor models, being cultured in Dulbecco’s Modified Eagles Medium (DMEM) (41965-039, Gibco, Life Technologies), supplemented with 10% fetal bovine serum (FBS) (S 0615, Merck), 1% Antibiotic-Antimycotic (AA; P06-07300, PAN Biotech) and 50 µg/mL Gentamicin (15750-060, Gibco, Life Technologies). Cell cultures were maintained at 37°C in a humidified environment of 5% CO_2_. Cells were detached with 0.05% Trypsin-EDTA 1 × (25300-054, Invitrogen, Thermo Fisher Scientific) at 37°C for approximately 5 min and split to new plates according to the experimental procedures.

Regarding experimental conditions, cells were cultured with 15 μM hydrogen peroxide (H_2_O_2_; 1.07210.0250, Merck), as a ROS generator, 1.5 μM Erastin (E7781, Sigma) as a ferroptosis inducer, 100 μM Propranolol (P8688, Sigma Aldrich) and 160 and 200 μM SeChry@PURE_G4_, for 6 and 16 h.

### Cell Death Analysis by Flow Cytometry

To analyze the effects of Propranolol, Erastin and SeChry on cell death, HUVECs (5×10^4^ cells/well) and MDA-MB-231 (5×10^4^ cells/well) were seeded in 24-well plates. After exposure to the experimental conditions, supernatants and cells were collected and centrifuged at 155 × *g* for 5 min. Cell pellets were incubated with 0.5 μL FITC-labeled Annexin V (640906, BioLegend) in Annexin V binding buffer 1× (10 mM Hepes (pH 7.4) (391333, Millipore), 0.14 M sodium chloride (NaCl; 106404, Merck), 2.5 mM calcium chloride (CaCl_2_; 449709, Sigma Aldrich) and incubated at room temperature for 15 min, in the dark. After incubation, cells were rinsed in 200 μL PBS 1×/0.1% (v/w) bovine serum albumin (BSA) and centrifuged at 155 × *g* for 2 min. The remaining pellet was resuspended in 200 μL of annexin V binding buffer 1× and 2.5 μL of 50 μg/mL propidium iodide (PI; P4170, Sigma Aldrich Aldrich) and analyzed by flow cytometry (FACScalibur – Becton Dickinson). *FlowJo X* v10.0.7 software (https://www.flowjo.com/) was used to analyze data.

### Wound Healing Assay

Cells were plated in 24-well plates (1×10^5^ cells/well) until the formation of a confluent monolayer. Once confluent, cells were incubated for 3 h with 5 μg/mL mitomycin-C (M4287, Sigma Aldrich) and a linear scratch in each monolayer was made with a P200 pipette tip, creating a wound across the well diameter. The media was replaced to remove debris and cells in suspension and the experimental conditions were added. Bright-field images of each well were acquired on the Olympus IX53 Inverted Microscope at the following timepoints: 0, 2, 4, 6, 8, 10 and 24 h. The wound closure was quantified using the *ImageJ* software (imagej.nih.gov/ij/).

### Tube-Forming Assay

A 48-well plate was coated with 100 µL matrigel (354230, Corning) and incubated at 37°C for 30 min until solidification. HUVECs were incubated with 2 μg/mL calcein (C1430, Invitrogen), a fluorescent cell permeable dye, for 30 min at 37°C and 5% CO_2_ and seeded (3×10^4^ cells/well) on the top of matrigel. Cells were exposed to the experimental conditions for 6h and representative images of the formed tube-like structures were acquired on an Olympus IX53 Inverted Microscope and analyzed with *ImageJ* software (imagej.nih.gov/ij/). The density of vessel-like structures formation (branch points number/μm^2^) was calculated as representative of vascular density.

### Reactive Oxygen Species (ROS) Quantification by Flow Cytometry

HUVECs (5×10^4^ cells/well) and MDA-MB-231 cells (5×10^4^ cells/well) were plated in 24-well plates. The intracellular ROS were detected in cells incubated with 10 μM DCF-DA probe (D6883, Sigma Aldrich) and mitochondrial ROS were detected in cells incubated with 5 μM MitoSox Red probe (M36008, Invitrogen), both at 37°C for 30 min. The acquisition was performed with FACScalibur (Becton Dickinson) and data were analyzed with *FlowJo X* v10.0.7 software (https://www.flowjo.com/).

### Lipid Peroxides Quantification by Flow Cytometry

HUVECs (5×10^4^ cells/well) were plated in 24-well plates. After experimental conditions, cells were incubated with 2 μM C11-Bodipy 581/591 (D3861, Invitrogen), for 30 min at 37°C in the dark. The excess dye was removed by washing with 2% FBS-1X PBS and cell pellets were resuspended in 2% FBS-1× PBS for the acquisition by flow cytometry (FACScalibur – Becton Dickinson). *FlowJo X* v10.0.7 software (https://www.flowjo.com/) was used to analyze data.

### High-Performance Liquid Chromatography (HPLC)

HUVECs were plated in 6-well plates (2×10^5^ cells/well) and after collection, cell pellets were lysed with 0.01% triton-PBS and centrifuged at 20 000 *g*, for 10 min at 4 °C. The assessment of pools of cysteine and GSH (total availability, total free fraction, reduced free, protein bound and oxidized pools) of lysed cells and supernatants was performed according to (34) and adapted to cell culture. The cysteine (Cys) and glutathione (GSH) metabolites were separated on a reversed-Phase C18 LiChroCART 250-4 column (LiChrospher 100 RP-18, 5 µm, VWR, USA) on isocratic elution mode for 22 min, at a flow rate of 0.6 mL/min by HPLC system (Shimadzu Scientific Instruments Inc) with a fluorescence detector operating at excitation and emission wavelengths of 385 and 515 nm, respectively. The mobile phase consisted of 100 mM acetate buffer (pH 4.5) and methanol (98:2 (v/v)). The concentrations of these thiols were normalized to the protein assessed with Bradford method (500-0006, Bio Rad). Results are presented as: Total GSH: levels of free form of oxidized or reduced GSH and GSH bound to proteins, it is the total pool of GSH in the cell; Free-total GSH: levels of free form of oxidized or reduced GSH, the total pool of GSH that is not bound to proteins; Free-reduced GSH: levels of reduced GSH free in the cell, and GSSG: levels of oxidized GSH free in the cell.

### Reverse Transcription and Quantifying PCR (RT-qPCR)

HUVECs (2×10^5^ cells/well) were plated in 6-well plates and after exposure to the experimental conditions, the RNA was extracted using RNeasy Mini Extraction kit (74104, Qiagen) and the cDNA synthesized from 1 µg RNA and reversely-transcribed by SuperScript II Reverse Transcriptase (18080e44, Invitrogen), both according to the manufacturer’s protocol. Quantitative Real-Time PCR was performed using Power SYBR Green PCR Master Mix (4367659, Applied Biosystems), according to manufacturer’s protocol and carried out in a LightCycler 480 instrument (Roche). The transcriptional expression of genes encoding prostaglandin-endoperoxide synthase 2 (*PTGS2*), glutathione peroxidase 4 (*GPX4*) and glutathione synthetase (*GSS*) was evaluated, using the primers: *PTGS2* (Fwd: CTGGCAGGGTTGCTGGTG; Rev: CATCTGCCTGCTCTGGTC); *GPX4* (Fwd: GCAGGAGCCAGGGAGTAAC; Rev: CCTTGGGTTGGATCTTCATCC), and *GSS* (Fwd: GAGAGAGGGTGGAGGTAAC; Rev: CCATGAGGATGTAGGAGGCC). Hypoxanthine-guanine phosphoribosyltransferase (HPRT; Fwd: TGACACTGGCAAAACAATGCA; Rev: GGTCCTTTTCACCAGCAAGCT) was used as housekeeping gene.

### Quantification of H_2_S in Cell Homogenates

HUVECs (2×10^5^ cells/well) were seeded in 6-well plates and cultured under the experimental conditions, for 16 h. After, cells were scrapped in PBS 1× and centrifuged at 210 × *g* for 5 min. The cell pellet was homogenized in NP40 lysis buffer (1% NP40, 150 mM NaCl, 50 mM Tris-Cl, pH 8.0) on ice for 30 min and centrifuged for 5 min at 20,000 × *g* 4°C. Cell homogenates (20 µL) were incubated in black 96-well plates with 80 µL of 10 µM 7-Azido-4-Methylcoumarin probe (AzMC, L511455, Sigma Aldrich). The protein concentration was determined with Bradford method using protein assay dye reagent concentrate (500-0006, Bio Rad). The H_2_S measurements were posteriorly normalized to the total protein concentration and to a blank sample (cellular lysates without probe). H_2_S production was monitored following fluorescent signal of AzMC probe (355 nm/460 nm) every 30 min for 2 h, in a VIKTOR3 instrument from *PerkinElmer/Wallac 1420 v3.0 software*.

### Immunofluorescence

For Ki67, ICAM and VCAM immunodetection, HUVECs (5×10^4^ cells/well) were cultured on glass slides with 0.2% gelatin coating and fixed in 2% paraformaldehyde, for 15 min at 4°C. After blocking with 1% BSA-1× PBS, cells were incubated with primary antibodies (anti-Ki67, 1:100 in 1% BSA-0.1% triton X-100- 1× PBS (w/v/v); sc-15402, Santa Cruz; anti-ICAM and anti-VCAM, 1:500 in 0.1% BSA-0.1% triton X-100-PBS (w/v/v); SRC023, Millipore), overnight at 4°C, followed by an incubation with secondary antibodies (Alexa Fluor 488 goat anti-rabbit, 1:1000 in 1% BSA-0.1% triton X-100-PBS; A-11078, Invitrogen - Thermo Fisher Scientific; Alexa Fluor 488 goat anti-mouse;115-545-003, Thermo Fisher Scientific and Alexa Fluor 594 donkey anti-mouse; A21203, Thermo Fisher Scientific both at 1:1000 in 0.1% BSA-0.1% triton X-100-PBS), for 2 h at room temperature.

For the cystine/glutamate antiporter system xc- (xCT) immunodetection, after fixation cell were incubated with 50 mM ammonium chloride (NH_4_Cl) for 10 min, followed by blocking and incubation with anti-xCT (1:100 in 0.5% BSA-0.1% saponin-PBS (w/v/v); ab1756, Millipore), for 30 min at room temperature.

For VE-Cadherin (VE-Cad) immunodetection, HUVECs (1×10^5^ cells/well) were cultured in 24-well plate with glass slides coated with 0.2% gelatin, until the formation of a confluent monolayer. After fixation and blocking, cells were incubated anti-VE-Cad (1:50 in 3% BSA-0.1% triton X-100-PBS (w/v/v); AF938, R&D), for 2 h at room temperature, followed by an incubation with the secondary antibody (Alexa Fluor 488 donkey anti-goat, 1:500 in 3% BSA-0.1% triton X-100-PBS; A11055, Thermo Fisher Scientific), for 2 h at room temperature.

All slides were mounted in VECTASHIELD media with DAPI (4′-6-diamidino-2-phenylindole; H-1200, Vector Labs) and examined by standard fluorescence microscopy, using an Axio Imager.Z1 microscope (Zeiss) with a *CytoVision^®^ software*.

The determination of cell proliferation rate was based on the ratio of total and Ki67^+^ nuclei and the quantification of ICAM and VCAM expression per cell was calculated according to the formula CTCF (corrected total cell fluorescence) = integrated density – (area of selected cell × mean fluorescence of background reading), both using *ImageJ software (imagej.nih.gov/ij/*).

### Cancer Cells Endothelial Adhesion

In a 24-well plate, calcein labelled-MDA-MB-231 (5×10^4^ cells/well) were seeded on the top of a HUVECs (1×10^5^ cells/well) monolayer pretreated with 100 ng/mL TNFα (H8916, Sigma), for 24 h. MDA-MB-231 were incubated with HUVECs (exposed previously to TNFα and experimental conditions) for 40 min, at 37°C in a humidified environment of 5% CO_2_. The non-adherent cells were removed by washing with PBS1X and images were acquired on an Olympus IX53 Inverted Microscope and analyzed using *ImageJ* software (*imagej.nih.gov/ij/*). Three fields in each well were evaluated (10× magnification).

### Transendothelial Cancer Cells Migration

HUVECs (5×10^4^ cells/well) were plated in 8µm pore transwells (upper wells) (3422, Corning) and exposed to 100 ng/mL TNFα for 24 h, and to experimental conditions for 16 h. MDA-MB-231 previously plated under starvation using serum-free DMEM, for 24 h, were incubated with calcein (2 μg/mL) and seeded (1.5×10^4^ cells/well) in serum free DMEM on the top of the HUVECs monolayer, for 5 h. Complete media was added to the lower well and used as chemoattractant. Cells on the upper Transwell^®^ surface were removed with a cotton swab and the invading MDA-MB-231- calcein labeled cells were photographed on an Olympus IX53 Inverted Microscope. Three fields in each well were counted (10× magnification) using the *ImageJ software* (imagej.nih.gov/ij/).

### Monocytes Isolation, Culture and Characterization

Monocytes isolation from peripheral blood of healthy blood donors (IPOLFG-Ethical committee UIC-1137) and further cell characterization was performed as described by Lopes-Coelho et al. (35). Briefly, monocytes cultured for 4 days in colony-forming unit (CFU) medium (130-091-277, MACS Technology) and for 1 day in complete EBM-2 were incubated with von Willebrand factor (vWF; 1:500 in 0.5% BSA-0.1% saponin-PBS; A0082, Dako), for 60 min at 4°C with gentle shaking, followed by the incubation with Alexa Fluor 488 anti-rabbit, for 30 min at 4°C in the dark, with gentle shaking. H_2_O_2_ (15 μM) was used as a promoter of monocytes differentiation into ECs (35). vWF expression was detected by flow cytometry in a FACScalibur–Becton and data were analyzed using the *FlowJo X* v10.0.7 software (https://www.flowjo.com/).

### SeChry@PURE_G4_ Synthesis

Selenium-containing chrysin (SeChry) was synthesized following a reported protocol (36). After purification, the formation of the product was confirmed by ^1^H NMR. ^1^H NMR (CDCl3, 400 MHz) δ (ppm): 7.96 (2H, d, *J*= 8.0 Hz), 7.76 (1H, s), 7.61 (1H, t, *J*= 8.0 Hz), 7.52 (2H, t, *J*= 8.0 Hz), 6.51 (1H, d, *J*= 4.0 Hz), 6.46 (1H, d, *J*= 4.0 Hz). Polyurea dendrimer generation four (PURE_G4_) was obtained using our supercritical-assisted polymerization protocol (37). SeChry was encapsulated in PURE_G4_ nanoparticles following our protocol (38). Briefly, SeChry (6.5 mg) was added to an aqueous solution (10 mL) of PURE_G4_ (125 mg) and stirred overnight. Then, the aqueous solution was extracted with CHCl_3_ to remove non-encapsulated or surface bound SeChry. No SeChry was found in the CHCl_3_ extracts (control by thin layer chromatography, TLC), thus confirming a full encapsulation. The release profile follows the usual profile reported for this nanodelivery system (39, 40).

### Statistical Analysis

All data were analyzed using student’s t-test, one-way ANOVA or two-way ANOVA in *GraphPad Prism* v7 software (www.graphpad.com/ *).* The assays were performed with at least 3 biological replicates *per* condition and the differences were determined statistically significant at *p* value < 0.05.

## Results

### A Ferroptosis-Like Mechanism Induced by Erastin Promotes ECs Activation, Which Is Impaired by Propranolol Through the Increase of H_2_S Levels

Erastin was used as a ferroptosis activator since it inhibits xCT (encoded by *SLC7A11*), which is responsible for the import of cystine (41, 42), the main source of cysteine to sustain GSH synthesis (43). xCT inhibition, impairs GPX4 and consequently leads to an increase in lipid peroxidation that is mediated by free active iron (44), and further ferroptosis (45).

**Figure 1:**
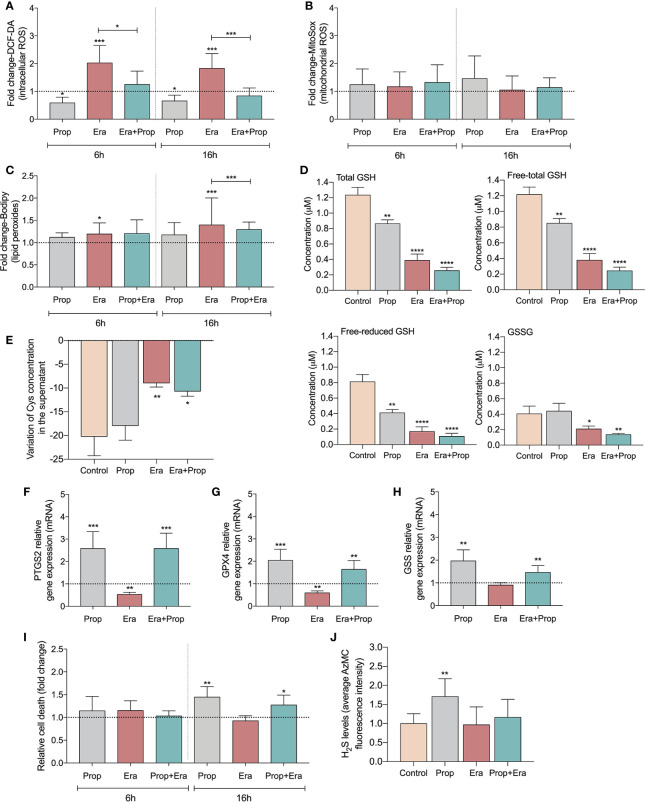
Erastin (Era) promotes increased levels of ROS-induced lipid peroxides and Propranolol (Prop), through the generation of hydrogen sulfide (H_2_S), is able to revert it. **(A)** The levels of intracellular ROS (DCF-DA) decrease upon Prop exposure, and Era, although increasing the ROS levels, when co-administrated with Prop the levels are similar to the control, at 6 and 16h. **(B)** The levels of mitochondrial ROS, assessed by MitoSox, are not affected by the presence of Era and/or Prop, at 6 and 16h. **(C)** Era induces lipid peroxides (C11-Bodipy) generation and although Prop alone did not affect the lipid peroxides content, its combination with Era reverts the levels generated by Era, being this effect more prominent at 16h. **(D)** The levels of GSH (total and LT:free total) decrease upon exposure to Era and/or Prop for 16h. **(E)** The variation of the extracellular levels of cysteine (Cys) indicate that Era inhibits the uptake of Cys by HUVECs, while Prop does not interfere with this process. **(F–H)** Show the regulation of transcriptional expression of genes encoding, respectively, prostaglandin-endoperoxide synthase 2 (*PTGS2*), glutathione peroxidase 4 (*GPX4*) and glutathione synthetase (*GSS*). Erastin decreases significantly GPX4 and PTGS2 expression and tend to decrease GSS expression, being this effect rescued by propranolol. **(I)** Era does not affect HUVECs death (annexin V plus PI positive cells) while 16h of Prop exposure, with and without Era, increases the ratio of HUVECs death. **(J)** Era does not affect H_2_S levels of HUVECs while Prop increases, at 16h. In graphs the dashed line represents the control condition. All data are normalized to the control condition and represented as mean ± SD. *p<0.5, **p<0.01, ***p<0.001, ****p<0.0001.

After confirming the expression of xCT in HUVECs (**Figure S1A**) and upon Erastin administration the ferroptosis-related features were analyzed. HUVECs exposed to Erastin presented an increase in intracellular levels of ROS (**Figure 1A**) and lipid peroxide levels (**Figure 1C**), without affecting mitochondrial ROS (**Figure 1B**) but decreasing the GSH levels (**Figure 1D**). Moreover, the inhibitory effect of Erastin in cyst(e)ine uptake was confirmed, since cells exposed to Erastin presented high levels of total cysteine (free oxidized+free reduced+protein bound) in culture medium (**Figure 1E**). Interestingly, after Erastin exposure the increased generation of ROS (**Figure 1A**) and lipid peroxides (**Figure 1C**), the depletion of GSH (**Figure 1D**) and the downregulation of genes involved in the scavenging of lipid peroxides (**Figures 1F, G**) and GSH synthesis (**Figure 1H**), did not promote ferroptosis-induced ECs death (**Figure 1I**). Moreover, we observed that Propranolol decreased intracellular ROS levels (**Figure 1A**) and also reverted the levels of ROS (**Figure 1A**) and lipid peroxides induced by Erastin (**Figure 1C**). Accordingly, Propranolol, alone or in combination with Erastin, decreased the levels of free (oxidized + reduced) and total GSH, maintaining the oxidized GSH levels similar to the control condition (**Figure 1D**).

Since GSH levels were not increased, we hypothesized that hydrogen sulfide (H_2_S) generation might underlie the antioxidant properties of Propranolol. H_2_S, a product of cysteine degradation (46, 47), is a powerful antioxidant (48) with a reduction potential similar to the couple glutathione disulfide/glutathione (GSSG/GSH) (49). There was an increase in H_2_S production upon Propranolol administration, while Erastin did not affect H_2_S levels (**Figure 1J**). This suggests that Propranolol favors cysteine flux for catabolism and not for GSH synthesis. Moreover, when Propranolol was combined with Erastin, the levels of H_2_S were similar to the control condition. Together, these results suggest that Propranolol’s antioxidant effect is mediated by H_2_S.

**Figure 2:**
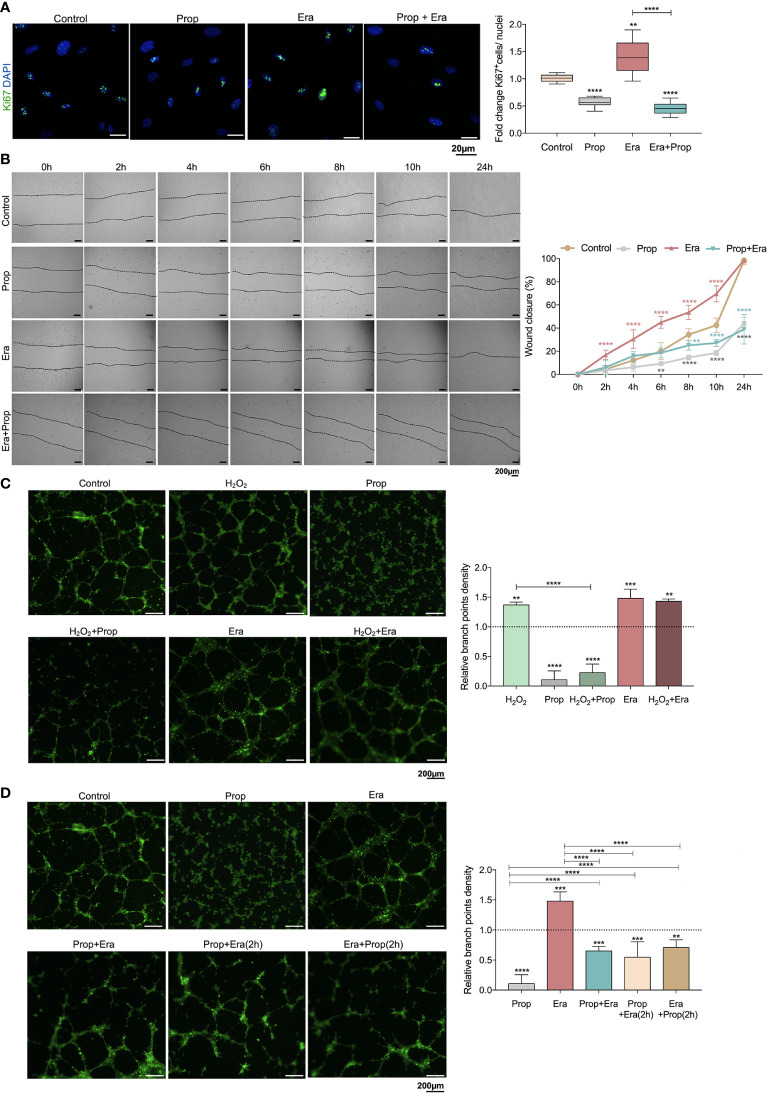
The ferroptosis-like mechanism driven by Erastin (Era) promotes endothelial cell (ECs) activation and Propranolol (Prop) impairs the phenotype induced by Era exposure. **(A)** The ferroptosis-like mechanism, generated by Era exposure, promotes HUVECs proliferation (increased ratio of Ki67^+^ (green) nuclei/total nuclei), while Prop decreases the rate of HUVECs proliferation and impairs the phenotype induced by Era. The panel shows representative microscope images of the Ki67 staining. **(B)** Era fosters HUVECs migration (increased % wound closure) and Prop inhibits and reverts the phenotype induced by Era. **(C, D)** Era increases the branch point density of vessel-like structures (proxy for vascular density) at the same range of H_2_O_2_ (ROS; positive control), with no additive effect. Prop, besides the impairment of vessel-like structures formation (decreased branch point density), inhibits the phenotype induced by Era, even when Prop is added to the vessel-like structures already formed in the presence of Era (Era+Prop (2h)) and contrariwise (Prop+Era (2h)). In graphs the dashed line represents the control condition. All data are normalized to the control condition and represented as mean ± SD. **p<0.01, ***p<0.001, ****p<0.0001.

Afterwards, we investigated if the generation of ROS-induced lipid peroxides promoted ECs activation, through this ferroptosis-like mechanism. It was observed that Erastin exposure increased HUVECs proliferation (**Figure 2A**) and migration (**Figure 2B**), suggesting that the ferroptosis-like mechanism has a role in the promotion of ECs activation, without affecting cell viability (**Figure 1I**).

Considering that Propranolol acts as an inhibitor of angiogenesis and ferroptosis, its interference with ferroptosis-mediated angiogenesis was also evaluated. In fact, HUVECs exposed to Propranolol showed decreased proliferation (**Figure 2A**) and migration (**Figure 2B**) and increased cell death, at 16h (**Figure 1I**). Moreover, Propranolol was able to revert the Erastin effect at 16h, leading to the inhibition of HUVECs proliferation (**Figure 2A**) and migration (**Figure 2B**), as well as disturbing cell viability (**Figure 1I**).

Since the pro-oxidative microenvironment promotes angiogenesis (6, 7), the HUVECs capacity to form vessels-like structures was tested under oxidative conditions, in an *in vitro* tube-forming assay, upon H_2_O_2_ exposure. Erastin exposure increased the branch points density at the same range of H_2_O_2_, and no cumulative effects between Erastin and H_2_O_2_ were observed (**Figure 2C**). Moreover, Propranolol decreased the vessel-like structure formation (**Figure 2C**) and reverted the stimulation by H_2_O_2_ and Erastin (**Figure 2C**). This effect of Propranolol was observed even when Propranolol was added to the vessel-like structures already formed in the presence of Erastin (**Figure 2D**). These results indicate that the Propranolol anti-angiogenic effect is related, at least in part, to the abrogation of the ferroptosis-like mechanism induced by Erastin.

### A Ferroptosis-Like Mechanism Induced by Erastin Promotes the Generation of Leakier ECs Structures Which Are Normalized by Propranolol, Blocking Cancer Cell Adhesion and Transendothelial Migration

**Figure 3:**
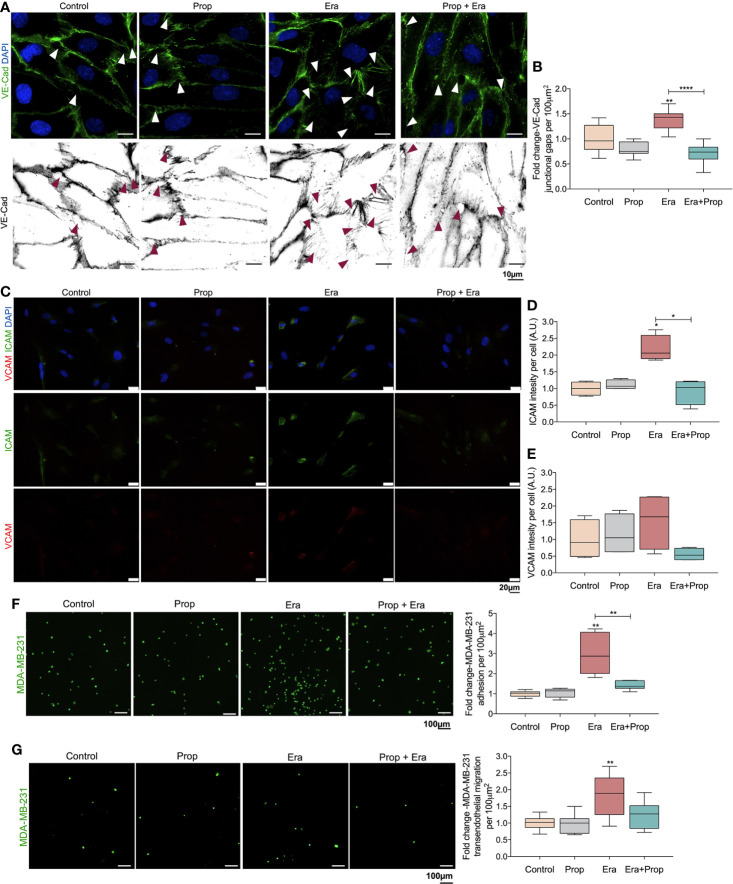
Erastin (Era) promotes the generation of a leakier EC monolayer while increases cancer cell-EC interaction and transendothelial migration. **(A)** The ferroptosis-like mechanism driven by Era promotes an increased generation of intercellular VE-Cadherin (VE-Cad) gaps per 100µm^2^, while Propranolol (Prop) is able to revert this phenotype. The panel shows representative images (scale: 10 µm) of VE-Cadherin (green) intercellular junctional gaps (arrows) in HUVECs exposed to Era and/or Prop for 16h. **(B)** Quantification of VE-Cad junctional gaps. **(C)** Immunofluorescence for ICAM and VCAM detection. **(D)** ICAM intensity per cell (HUVECs; A.U.: arbitrary units) increases upon Era exposure and although Prop alone does not affect ICAM expression, it is able to abrogate the expression induced by Era, at 16h. **(E)** VCAM intensity per cell (A.U.: arbitrary units) shows a tendency to increase under Era exposure. The ferroptosis-like mechanism, induced by Era, promotes cancer cell (MDA-MB-231-calcein labelled cells) adhesion to HUVECs **(F)** and transendothelial migration **(G)**. Prop alone has no effect but impairs cancer cell adhesion and transendothelial migration induced by Era. The panels show representative microscope images (scale: 100 µm; MDA-MB-231-calcein labelled cells (green)). All data are normalized to the control condition and represented as mean ± SD. *p<0.05, **p<0.01, ***p<0.001.

The generation of a pro-oxidative microenvironment is correlated with the formation of disorganized and leakier vessel networks (5). Thus, the adhesion structures in HUVECs were evaluated, through VE-Cad immunodetection. VE-Cad is a component of ECs adherents junctions, crucial for the stability and function of the mature vessels (50). Erastin affected the HUVECs monolayer stability, by increasing VE-Cad intercellular junctional gaps, and Propranolol reverted this effect (**Figures 3A, B**). This indicates that the proliferation of ECs is mediated by a ferroptosis like-mechanism (**Figure 2A**), although they form more instable structures due to increased intercellular junctional gaps between ECs (**Figures 3A, B**). Interestingly, Propranolol reverted this phenotype (**Figures 3A, B**), inducing the stabilization of the ECs monolayer.

ICAM and VCAM adhesion molecules are important for cancer cell-EC interaction during the metastatic cascade (51–53), thus, we evaluated their expression. HUVECs exposed to Erastin significantly increased ICAM expression, as well as VCAM expression, although not in a statistically significant level (**Figures 3C–E**). On the contrary, Propranolol alone did not alter ICAM and VCAM expression, but it reverted the effect of Erastin by decreasing ICAM and VCAM levels (**Figures 3C–E**).

Considering the increased expression of ICAM (**Figures 3C, D**), we have also explored the effect of Erastin and Propranolol in the adhesion of cancer cells to ECs and in transendothelial migration. The triple negative breast cancer cell line MDA-MB-231 was co-cultured on the top of a previously established HUVECs monolayer exposed to Erastin and/or Propranolol. Erastin increased the number of cancer cells adherent to the ECs monolayer and stimulated transendothelial migration, while Propranolol reverted both Erastin effects (**Figures 3F, G**). Our results indicate that a ferroptosis-like mechanism has also a role in the promotion of vessel permeability and in cancer cell adhesion and extravasation. Summing up, Propranolol was able to revert the phenotype induced by Erastin, decreasing the intercellular junctional gaps (**Figures 3A, B**), the cancer cell adhesion to ECs (**Figure 3F**) and transendothelial migration (**Figure 3G**), therefore suggesting that Propranolol could eventually impair, or at least retard, the metastatic process (17).

### Neither Erastin nor Propranolol Affect Monocytes (EPCs) Differentiation in ECs

**Figure 4:**
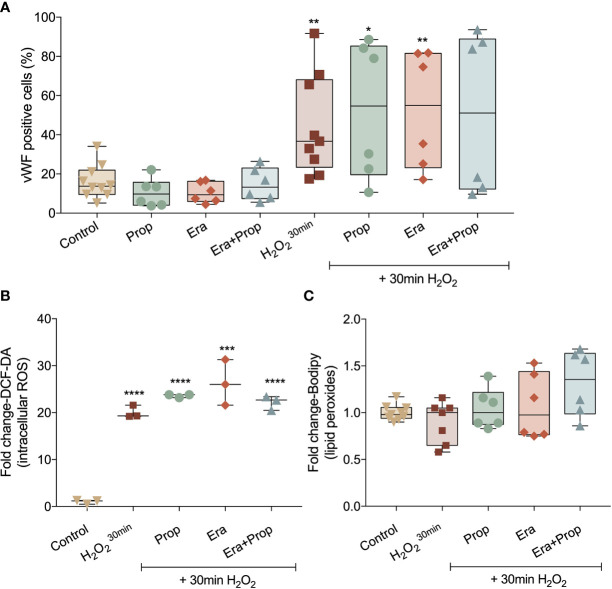
Erastin (Era) and Propranolol (Prop) do not affect the differentiation route of monocytes into ECs. **(A)** Neither Era and/or Prop affect the expression of vWF, even when co-exposed with a short H_2_O_2_ stimulation (positive control of the differentiation pattern of monocytes), indicating that Era and/or Prop have no impact in the differentiation process of monocytes-derived cells into ECs. Era and/or Prop exposure before the short H_2_O_2_ stimulation does not influence the intracellular ROS (DCF-DA; **B**) and lipid peroxide (11C-Bodipy; **C**) levels. In **(B, C)** data are normalized to the control condition and represented as mean ± SD. *p<0.5, **p<0.01, ***p<0.001, ****p<0.0001.

Recently our group disclosed that monocytes act as endothelial progenitor cells and their differentiation into ECs and incorporation in blood vessels depend on a ROS-enriched microenvironment (35). Considering the effect of a ferroptosis-like mechanism in ECs activation, we explored its impact on the differentiation route of monocytes into ECs, assessed by the gain of von Willebrand factor (vWF). Erastin and/or Propranolol exposure had no effect in vWF expression and their concomitant exposure with a H_2_O_2_ stimulation (positive control of the differentiation pattern of monocytes) did not affect the vWF expression stimulated by H_2_O_2_ (**Figure 4A**). Moreover, ROS and lipid peroxide (**Figures 4B, C**) levels did not alter with Erastin exposure after H_2_O_2_ stimulation. In contrast to ECs, Propranolol did not affect ROS and lipid peroxide levels in monocytes (**Figures 4B, C**), suggesting that the antioxidant role of Propranolol verified during ECs activation did not interfere with the differentiation process of monocytes towards ECs.

### Sechry@PURE_G4_ Plus Propranolol: A Putative Anti-Cancer Strategy, Acting on Both Cancer Cell Death and ECs Hyperactivation Prevention

The anti-angiogenic therapy to treat cancer has an efficiency far from the expected. Thus, a promising therapeutic approach would be a dual-effect therapy, which is cytotoxic to cancer cells and stabilizes the vessels, in order to improve the delivery of chemotherapy to the tumor.

**Figure 5:**
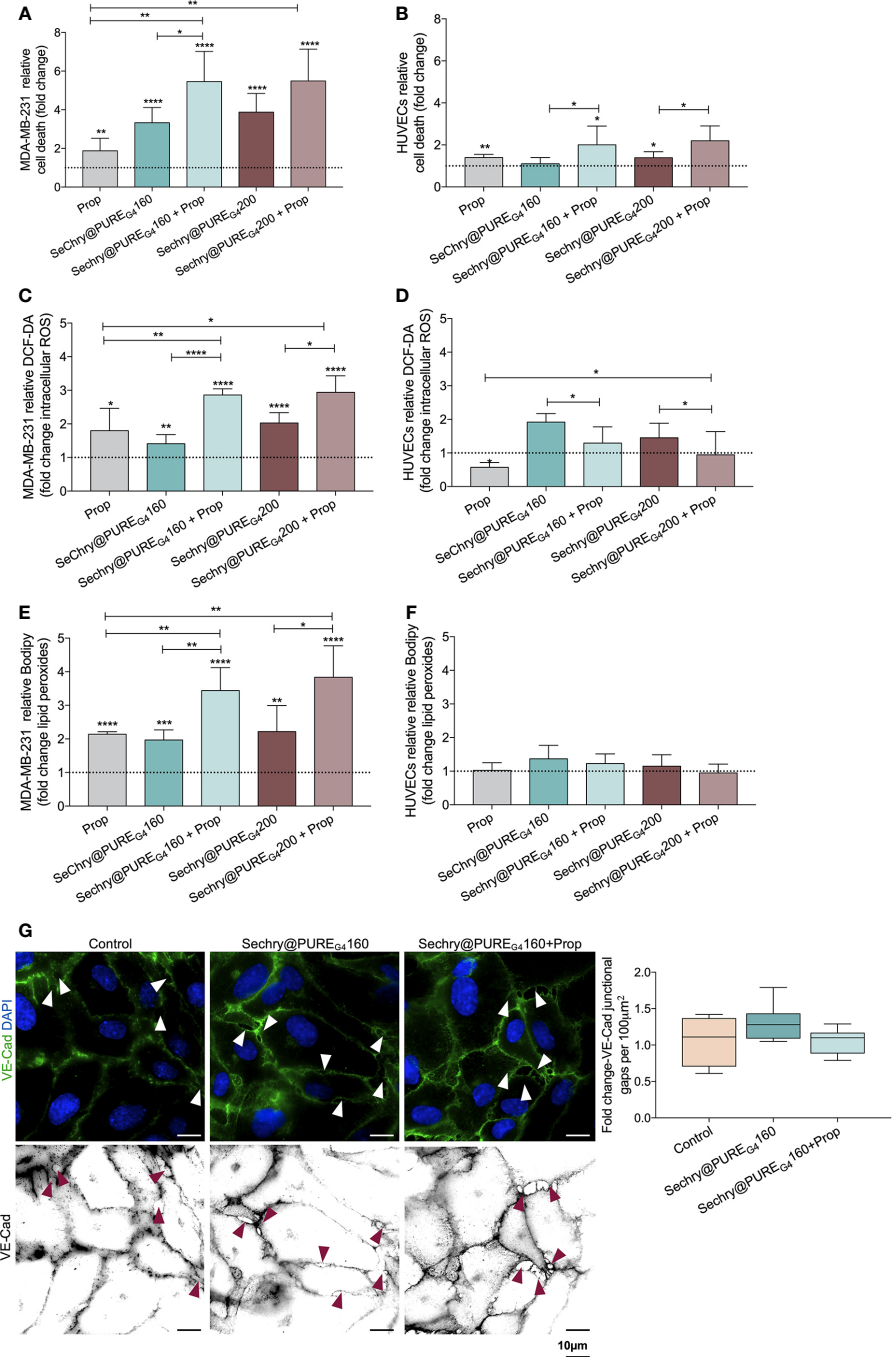
SeChry@PUREG_4_ plus Propranolol (Prop) increases cancer cell death through the generation of oxidative stress, while in ECs Prop acts as an antioxidant, reverting ROS levels induced by SeChry@PUREG_4_. **(A)** SeChry@PUREG_4_ (160 µM and 200 µM) exposure promotes cancer cell death (MDA-MB-231), being this effect boosted by Prop. **(B)** ECs (HUVECs) are more resistant to SeChry@PUREG_4_ -induced cell death, even under Prop exposure. **(C–F)** Contrarily to HUVECs, in MDA-MB-231 Prop alone increases **(C, D)** intracellular ROS (DCF-DA) and **(E, F)** lipid peroxide (11C-Bodipy) levels and it does not revert the generation of ROS-induced lipid peroxidation induced by SeChry@PUREG_4_. **(G)** SeChry@PUREG_4_ does not impact the generation of VE-Cad intercellular junctional gaps. The panel shows representative images (scale: 10 µm) of VE-Cadherin (green) intercellular junctional gaps (arrows). In graphs the dashed line represents the control condition. All data are normalized to the control condition and represented as mean ± SD. *p<0.05, **p<0.01, ***p<0.001, ****p<0.0001.

Recently, our team showed that SeChry@PURE_G4_ nanoformulation had an anti-tumoral effect thereby depleting GSH and inhibiting the H_2_S producing enzyme, cystathionine beta synthase (CBS) (38). Thus, SeChry@PURE_G4_ is a good candidate to be tested with Propranolol, in a dual-effect therapy, inducing cancer cell death and stabilizing tumor vessels. Hence, we investigated the SeChry@PURE_G4_ effect with and without Propranolol on MDA-MB-231 and HUVECs. Interestingly, MDA-MB-231 were more sensitive to Propranolol exposure than ECs (**Figures 5A** and **1I**), increasing MDA-MB-231 cell death by 2-fold (**Figure 5A**). The two tested concentrations of SeChry@PURE_G4_ (160 and 200 μM) promoted cancer cell death, mainly when combined with Propranolol (**Figure 5B**). Interestingly, HUVECs cell death was not altered by 160 μM SeChry@PURE_G4_ and a slight increase was observed with 200 μM SeChry@PURE_G4_ (**Figure 5B**). These results confirmed the anti-tumoral function of SeChry and indicate that Propranolol has distinct effects on the viability of cancer cells and ECs.

Regarding the effect SeChry@PURE_G4_ exposure on the intracellular ROS levels, while in cancer cells the combination of SeChry@PURE_G4_ and Propranolol induced an additive increase in ROS levels, in HUVECs the addition of Propranolol had the opposite effect, reinforcing the previously observed antioxidant role of Propranolol in ECs (**Figures 5C, D**). Accordingly, the ROS-induced lipid peroxide levels increased upon SeChry@PURE_G4_ with or without Propranolol exposure, in MDA-MB-231 (**Figure 5E**) but not in HUVECs (**Figure 5F**). Moreover, HUVECs exposed to SeChry@PURE_G4_ presented a more stable monolayer with reduced VE-Cad intercellular junctional gaps (**Figure 5G**).

Together, these results suggest that SeChry@PURE_G4_ plus Propranolol could be an interesting strategy for cancer treatment, targeting both cancer cells and ECs. In cancer cells, SeChry@PURE_G4_ and Propranolol would have anti-tumor effects through the promotion of cell death mediated by ferroptosis, whereas in ECs, Propranolol would impair oxidative stress-induced mechanisms, blunting ECs hyperactivation and promoting stability, which in turn might decrease transendothelial cancer cell migration and impair metastasis.

## Discussion

During tumorigenesis, the increased metabolic rate of cancer cells drives the generation of a pro-oxidative tumor microenvironment, responsible for the production and release of pro-angiogenic factors by cancer and tumor-associated stromal cells (54, 55). The ROS production within the tumor microenvironment is also stimulated by the main players of ECs activation, VEGF and hypoxia (56–59). The major production of ROS depends on NOX, which generate O_2_.^-^ through the transfer of electrons from NADPH to oxygen (56–59). The generation of NOX-dependent ROS increases VEGF secretion and angiogenesis, in a HIF1α-dependent manner (60). Therefore, the generation of a pro-oxidative and pro-angiogenic tumor microenvironment seems to work synergistically in the promotion of tumor angiogenesis.

Ferroptosis has been firstly described as an iron-dependent programed cell death characterized by the accumulation of lipid peroxides (61), although new evidences have shown that ferroptosis is not a strict cell death mechanism, being relevant in the regulation of biological and pathophysiological processes (9–11, 62–64). Ferroptosis-inducing compounds, as Erastin, affect the antioxidant capacity of cells through the inhibition of xCT, also expressed in ECs (HUVECs) (**Figure S1A**). The impairment of xCT activity disturbs cystine import, the main source of cysteine for GSH synthesis. GSH acts as an electron donor to reduce lipid hydroperoxides upon GPX4 action (9, 10, 65). Besides the role of GSH as a scavenger, the proteins *S*-glutathionylation should be explored in the future, since it seems to be a mechanism of redox switch in ECs accounting for vascular homeostasis (66). So far, there are no specific markers of ferroptosis as a cell death mechanism and because we were exploring the non-lethal effect of ferroptosis, in this work we evaluated the levels of ROS-induced lipids peroxides underlying ferroptosis and GSH dynamics (67). The expression of genes pointed as being decreased upon ferroptosis activation, *PTGS2*, *GPX4* and *GSS* was also assessed (10, 68–70). In our experimental conditions, we observed that Erastin significantly decreases cyst(e)ine uptake (**Figure 1E**) and promotes the increase of intracellular ROS (**Figure 1A**) and lipid peroxide levels (**Figure 1C**), without affecting the mitochondrial ROS content (**Figure 1B**). In agreement, the GSH depletion (**Figure 1D**) and the decreased expression of *PTGS2*, *GPX4* and *GSS* genes (**Figures 1F–H**) was observed upon Erastin exposure. All these ferroptosis-like features did not account for ECs death (**Figure 1I**).

The anti-ferroptotic property of Propranolol has been recently described, showing that the peroxyl radicals scavenging property is independent of β1-blockade activity (29). Here, we observed that Propranolol decreased reduced and total GSH levels, but it kept the GSSG (oxidized GSH) levels similar to control conditions (**Figure 1D**), as well as it decreased the intracellular ROS levels (**Figure 1A**) and reverted the accumulation of ROS-induced lipid peroxides induced by Erastin (**Figures 1A, C**). The expression of *PTGS2*, *GPX4* and *GSS* was rescued by Propranolol (**Figures 1F–H**). In fact, the increased H_2_S levels upon Propranolol exposure (**Figure 1J**), indicated that the antioxidant Propranolol property might be mediated by H_2_S, since it is a gasotransmitter capable of regulating oxidative stress by directly scavenging ROS (48, 71).

Considering that the generation of a pro-oxidative microenvironment is implicated in the promotion of the angiogenic switch and further angiogenesis (6, 7), we unraveled, for the first time, the role of the ferroptosis-like mechanism in ECs activation. We showed that the ferroptosis-like mechanism induces ECs hyperactivation, by increasing cell proliferation and migration (**Figures 2A, B**) and also promoting the formation of vessel-like structures (**Figure 2C**), mimicking the *in vivo* capacity of ECs to form blood vessels. Interestingly, Erastin stimulates the formation of vessel-like structures at the same range of H_2_O_2_ (ROS) (**Figure 2C**), supporting the involvement of the ferroptosis-like mechanism on angiogenic processes.

Propranolol acts as an inhibitor of angiogenesis and it was recently described to suppress proliferation, migration and tube formation of hemangioma cells through the HIF-1α-VEGF-A axis (72, 73) and to decrease the expression of angiogenic growth factors, as VEGF and FGF (20). However, the precise cellular mechanism underlying blood vessels disruption and angiogenesis impairment is still unknown. In this study, we observed that Propranolol, besides its anti-angiogenic effect under basal culture conditions, is able to abrogate the stimulation of ECs activation induced by Erastin and to disrupt already established vessel-like structures (**Figures 2A–D**), suggesting that the Propranolol anti-angiogenic effect is related, at least in part, to the abrogation of the ferroptosis-like mechanism.

The cancer-associated vasculature is characterized by an increased permeability and interstitial fluid pressure due to the disruption of ECs junctions, which reveals to be pivotal in cancer cell adhesion, intravasion and metastasis (74, 75). The increased VEGF levels, in the tumor microenvironment, promote VE-Cad adherens junction phosphorylation and internalization in ECs, leading to an impaired adhesiveness and non-functional vessels (74, 76). Besides the role of the ferroptosis-like mechanism on ECs activation, Erastin exposure promotes the formation of VE-Cad intercellular junctional gaps (**Figures 3A, B**), which results in the generation of a leakier ECs structure. This is accompanied by an increase in ICAM adhesion molecule expression (**Figures 3C, D**), a crucial protein for cancer cell and ECs interaction during the metastatic cascade (51–53). Increased ICAM expression (**Figures 3C, D**) promotes cancer cell adhesion to ECs and transendothelial migration (**Figures 3F, G**). Our results support that Propranolol contributes to vessel stabilization and putatively disturbs metastasis, since Propranolol did not alter VE-Cad intercellular junctional gaps (**Figures 3A, B**), ICAM expression and further cancer cell: ECs adhesion (**Figure 3F**) and transendothelial cancer cell migration (**Figure 3G**). Accordingly, Propranolol reverted the pro-metastatic ECs phenotype induced by Erastin, by decreasing VE-Cad intercellular junctional gaps (**Figures 3A, B**) and ICAM expression (**Figure 3C**); and by disturbing cancer cell adhesion to ECs and transendothelial cancer cell migration (**Figures 3F, G**). Therefore, the effect of the ferroptosis-like mechanism, at a non-lethal level, mimics the pathophysiological angiogenic process in cancer, characterized by an ECs hyperactivation that leads to the formation of a leakier vascular network (74, 75, 77, 78). On the other hand, Propranolol administration blocks the angiogenic switch, decelerating angiogenesis and further tumor growth, while preventing the generation of a leakier vasculature, decreasing metastasis and putatively increasing the delivery of cytotoxic drugs to the cancer cells.

Recently our group showed that monocytes act as endothelial progenitor cells that differentiate into ECs upon oxidative stress and are capable of incorporating into the tumor neo-vasculature, contributing to cancer progression (35). In this context, we explored if the pro-angiogenic stimuli pushing monocytes towards ECs differentiation, also benefited from a ferroptosis-like mechanism. In fact, we observed that neither Erastin nor Propranolol interfered with monocytes differentiation into ECs (**Figure 4**).

In cancer cells, selenium compounds interfere with the selenium uptake, selenocysteine biosynthesis and the production of selenoproteins, such as GPX4, consequently abrogating cell protection against ferroptosis (79). Moreover, selenium compounds display antioxidant or pro-oxidant properties, depending on their concentrations (80, 81); and they can be used as cytotoxic compounds showing anti-cancer properties and overcoming cisplatin resistance and multiple drug resistance (36, 38, 82, 83).

Considering the dual effect of these compounds and the antioxidant role of Propranolol in ECs, we explored if a combination of Propranolol with SeChry encapsulated in PURE_G4_ nanoparticles (SeChry@PURE_G4_) could be used as a strategy to simultaneously induce cancer cell death and stabilize ECs, resembling tumor vessels. Interestingly, in breast cancer cells (MDA-MB-231), Propranolol alone increased cell death by 2-fold (**Figure 5A**), demonstrating that, besides its role in preventing ECs hyperactivation, Propranolol could also promote cancer cells death. Moreover, MDA-MB-231 were more sensitive to SeChry@PURE_G4_ than HUVECs, showing higher cell death levels upon exposure with or without Propranolol (**Figures 5A, B**). These results reinforce the anti-tumoral SeChry@PURE_G4_ effect on ovarian cancer cells, recently described by us (38), and confirmed the differential effect of Propranolol on cancer cells and ECs. Interestingly, SeChry@PURE_G4_ exposure induced ferroptosis in cancer cells, as it increased the generation of intracellular ROS and the accumulation of lipid peroxides (**Figures 5C, E**), which ultimately promote cancer cell death (**Figure 5A**). Contrarily to the observations in HUVECs, in MDA-MB-231, Propranolol did not display antioxidant features, since the levels of ROS and ROS-induced lipid peroxides increased upon Propranolol exposure (**Figures 5C, E**). In HUVECs, SeChry@PURE_G4_ had no effect on the generation of lipid peroxides (**Figure 5F**) and did not affect the formation of VE-Cad junctional gaps (**Figure 5G**), suggesting that SeChry@PURE_G4_ does not compromise vessels stability. Together, these results demonstrate that SeChry@PURE_G4_ plus Propranolol administration is a promising strategy for cancer treatment, since this combination is able to induce cancer cell death through ferroptosis, while avoiding the formation of a leakier vasculature, which ultimately impairs cancer cell intravasation and metastasis.

## Conclusion

The ferroptosis-like mechanism, mediated by Erastin, through GSH depletion and ROS-induced lipid peroxide generation is implicated in the regulation of some pathophysiological ECs features, promoting ECs hyperactivation, leakiness and cancer cell migration. Propranolol scavenging activity mediated by H_2_S impairs the generation of oxidative stress, reverting the ECs phenotype observed under Erastin exposure. Additionally, despite the effects of the ferroptosis-like mechanism on ECs activation, it did not affect the differentiation process of monocytes into ECs.

**Figure 6:**
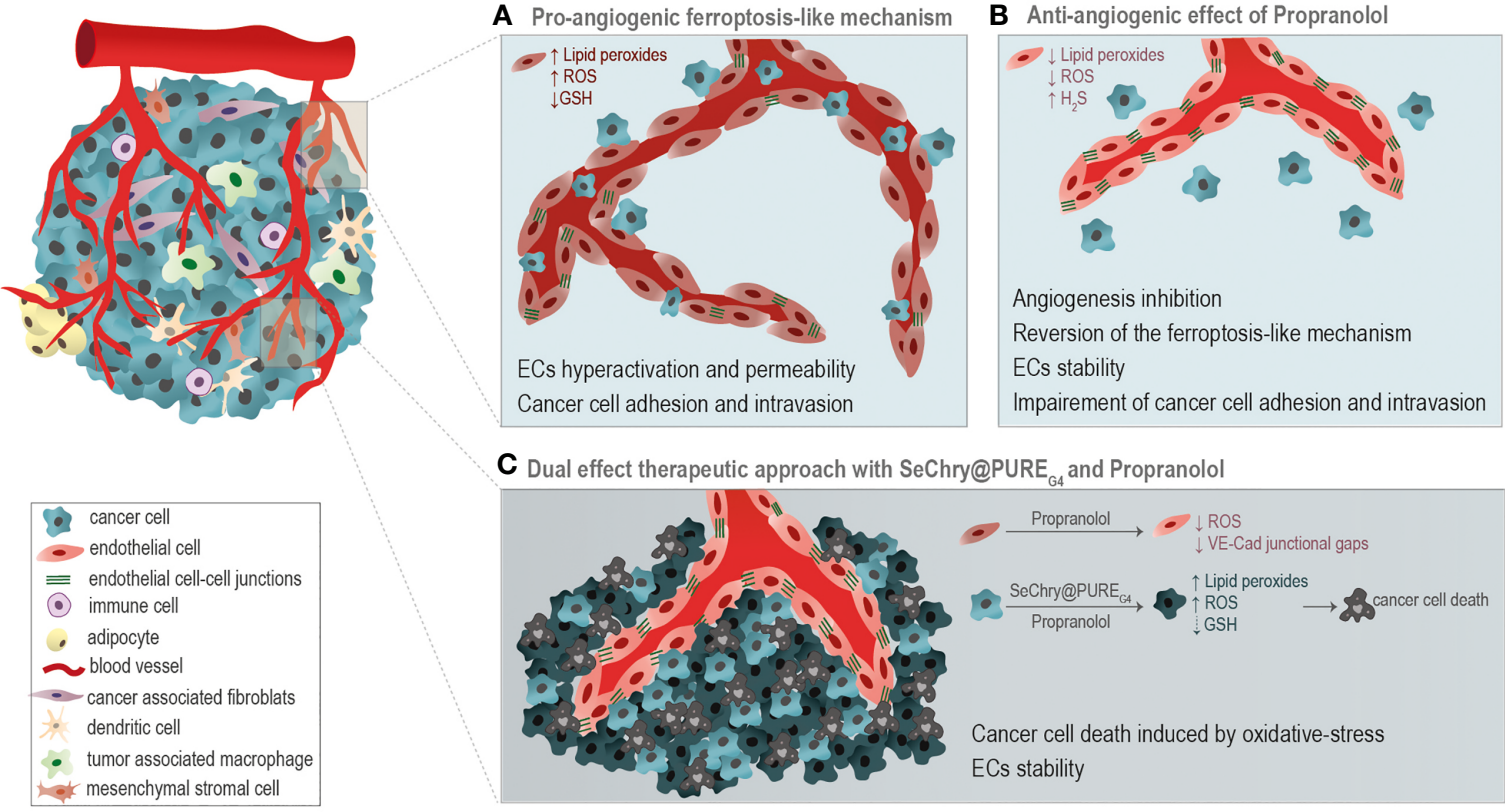
Taking advantage of the differential oxidative stress response of cancer cells and endothelial cells (ECs). **(A)** A pro-angiogenic ferroptosis-like mechanism, through the generation of ROS, accumulation of lipid peroxides and glutathione (GSH) depletion, is implicated in the promotion of ECs hyperactivation, vessels leakiness and cancer cell adhesion and intravasation. **(B)** Propranolol (Prop) ROS scavenging activity is anti-angiogenic, impairing ECs activation underlined by the ferroptosis-like mechanism. **(C)** The combination of SeChry@PURE_G4_ nanoparticles and Prop was unraveled as a potential cancer therapy. SeChry@PURE_G4_ induces cancer cell death mediated by pro-oxidative features, while Prop stabilizes ECs and prevents the formation of a leakier vasculature, avoiding metastasis. Prop enhances the pro-oxidative features of Sechry@PURE_G4_ effect.

Moreover, in this paper we disclose the potential use of SeChry@PURE_G4_, a selenium-containing nanoformulation, in combination with Propranolol, as a good strategy for cancer treatment. The combination of SeChry@PURE_G4_ with Propranolol induces cancer cell death mediated by pro-oxidant features, while in ECs it prevents the formation of a leakier vasculature (**Figure 6**), pivotal in cancer cell intravasation and metastasis.

## Data Availability Statement

The raw data supporting the conclusions of this article will be made available by the authors, without undue reservation.

## Author Contributions

FL-C-Planned, executed the majority of the experimental assays and wrote the first draft of the manuscript. FM, AH, CM and RP-Performed experimental assays and revised the manuscript. AA, VB and SP-Supervised the project and revised and discussed the manuscript. JS-Supervised the project, provided funding, and revised and discussed the manuscript. All authors contributed to the article and approved the submitted version

## Funding

The project was funded by IPOLFG EPE and by iNOVA4Health (UID/Multi/04462/2019) a program financially supported by *Fundação para a Ciência e Tecnologia (FCT)/Ministério da Educação e Ciência*, through national funds. We also acknowledge funding from FCT-MCTES through the project DREAM—PTDC/MEC-ONC/29327/2017. FL-C PhD fellowship was funded by FCT (PD/BD/128337/2017).

## Conflict of Interest

The authors declare that the research was conducted in the absence of any commercial or financial relationships that could be construed as a potential conflict of interest.

## Supplementary Material

Supplementary Figure 1 xCT is expressed in endothelial cells (HUVECs) and in comparison to cancer cells, are more sensitive to the generation of lipid peroxides induced by Erastin (Era). **(A)** HUVECs expresses xCT (green) mainly in mitochondria (TOM20, red), and its expression is not affected by Era and/or Propranolol (Prop), for 16 h (scale: 20μm). **(B)** HUVECs are more sensitive to the generation of Era-induced lipid peroxides than cancer cells (MDA-MB-231). Two Era concentrations were tested (1,5μM and 5 μM) during different time points (1, 6 and 16h). In graphs the dashed line represents the control condition. All data are normalized to the control condition and represented as mean ± SD. *p<0.05, **p<0.01, ***p<0.001.
