# Supplementary figures and images for: The Activation of Endothelial Cells Relies on a Ferroptosis-Like Mechanism: Novel Perspectives in Management of Angiogenesis and Cancer Therapy
Source: Front Oncol. 2021 May 10;11:656229. doi: 10.3389/fonc.2021.656229 (PMC8141735; doi:10.3389/fonc.2021.656229)

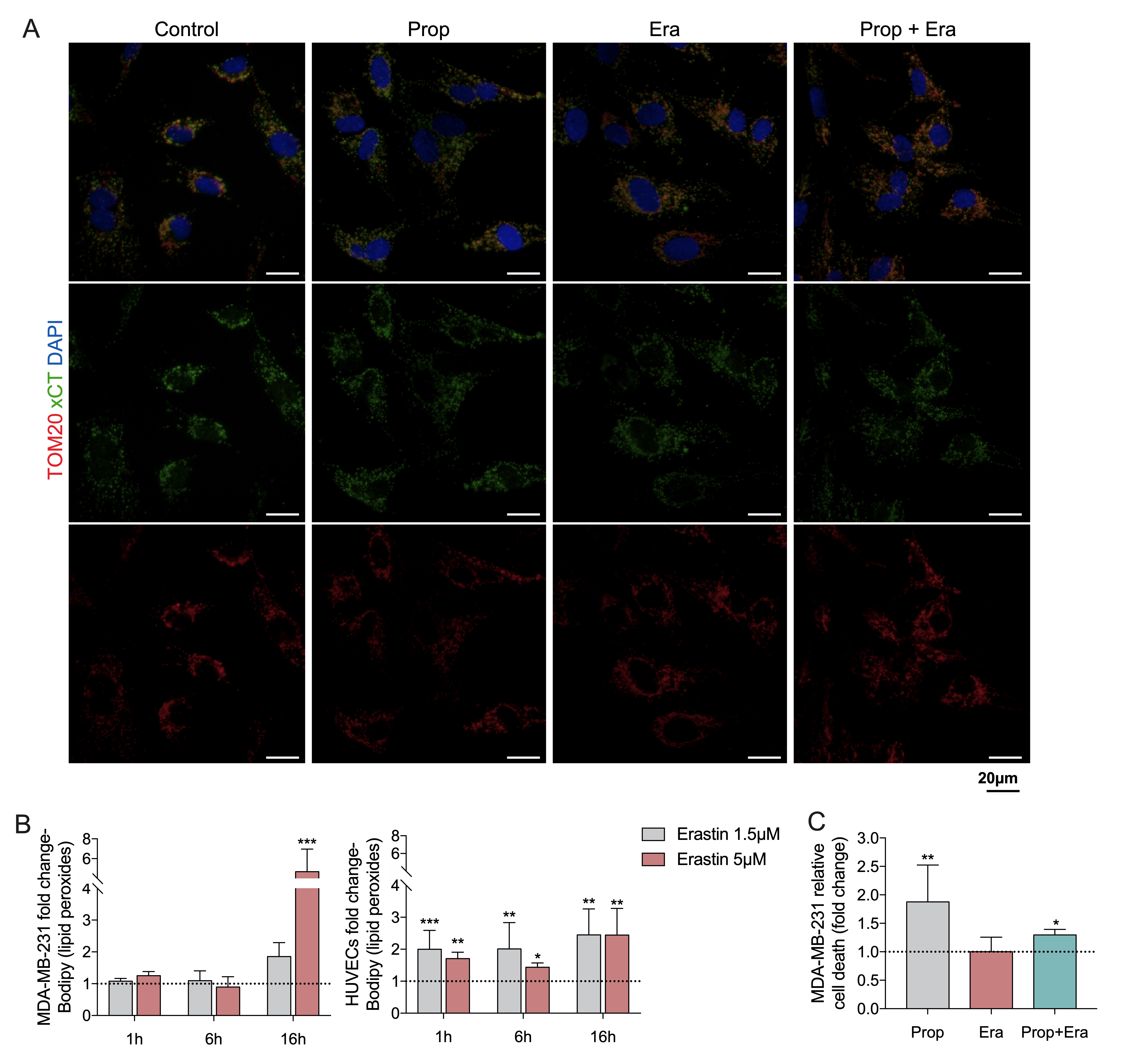

Supplement: Supplementary Figure 1 — xCT is expressed in endothelial cells (HUVECs) and in comparison to cancer cells, are more sensitive to the generation of lipid peroxides induced by Erastin (Era). (A) HUVECs expresses xCT (green) mainly in mitochondria (TOM20, red), and its expression is not affected by Era and/or Propranolol (Prop), for 16 h (scale: 20μm). (B) HUVECs are more sensitive to the generation of Era-induced lipid peroxides than cancer cells (MDA-MB-231). Two Era concentrations were tested (1,5μM and 5 μM) during different time points (1, 6 and 16h). In graphs the dashed line represents the control condition. All data are normalized to the control condition and represented as mean ± SD. *p<0.05, **p<0.01, ***p<0.001. [file Image_1.jpg]
